# pH-Sensitive Hydrogel for Micro-Fluidic Valve

**DOI:** 10.3390/jfb3030464

**Published:** 2012-07-10

**Authors:** Yan Zhang, Zishun Liu, Somsak Swaddiwudhipong, Haiyan Miao, Zhiwei Ding, Zhengzhi Yang

**Affiliations:** 1 Department of Civil and Environmental Engineering, National University of Singapore, Singapore 117576, Singapore; Email: u0904614@nus.edu.sg (Z.Y.); ceesomsa@nus.edu.sg (S.S.); 2 Institute of High Performance Computing, Agency of Science, Technology and Research, 1 Fusionopolis Way, #16-16 Connexis, Singapore 138632, Singapore 138632, Singapore; Email: miaohy@ihpc.a-star.edu.sg; 3 ICAM, State Key Laboratory for Mechanical Structure Strength and Vibration, Xi’an Jiaotong University, Xi’an 710049, China; 4 Engineering Science Programme, National University of Singapore, Singapore 117576, Singapore; Email: a0078004@nus.edu.sg (D.Z.W.); a0004672@nus.edu.sg (Y.Z.Z.)

**Keywords:** hydrogel, finite element, fluid structure interaction, micro-fluidic valve

## Abstract

The deformation behavior of a pH-sensitive hydrogel micro-fluidic valve system is investigated using inhomogeneous gel deformation theory, in which the fluid-structure interaction (FSI) of the gel solid and fluid flow in the pipe is considered. We use a finite element method with a well adopted hydrogel constitutive equation, which is coded in commercial software, ABAQUS, to simulate the hydrogel valve swelling deformation, while FLUENT is adopted to model the fluid flow in the pipe of the hydrogel valve system. The study demonstrates that FSI significantly affects the gel swelling deformed shapes, fluid flow pressure and velocity patterns. FSI has to be considered in the study on fluid flow regulated by hydrogel microfluidic valve. The study provides a more accurate and adoptable model for future design of new pH-sensitive hydrogel valves, and also gives a useful guideline for further studies on hydrogel fluidic applications.

## 1. Introduction

Hydrogel has become more popular recently as a functional engineering and/or bio-material. It has earned the reputation as a “smart material” or “intelligent gel”, due to its ability to change its volume or shape in response to a surrounding signal [1,2,3,4,5,6,7]. Hydrogel is a product of water-swollen, crosslinked polymers that absorb water in quantities up to thousands of times that of the dry network. In an aqueous environment, hydrogel will undergo a reversible phase transformation that results in striking volumetric change upon exposure and removal of a stimulus. The most important property of hydrogel is its stimulus-sensitivity depending on external conditions, including pH value, temperature, pressure, salt concentration etc. These conditions dramatically affect the swelling behavior, network structure, permeability and mechanical strength of hydrogels [8,9,10]. Hydrogel can be used in many fields due to its unique swelling and highly hydrated structure. The material can be used as a fluid pump [11], valve [5], and muscle-like actuator [12] it has also been used extensively for biomedical purposes, e.g. drug delivery systems, wound management and tissue engineering [13,14,15,16,17].

The micro fluidic valve is a device that regulates the flow of a fluid. It consists of a diaphragm or piston coupled to an actuator that controls the diaphragm to open or close a channel. The pH-sensitive hydrogel can be used in engineering applications as microfluidic valves to act as automatic sensors and actuators [2,18,19,20,21,22,23]. A conventional microfluidic valve requires an external power supply and is relatively complex in its assembly. Utilization of the pH-sensitive hydrogel enhances the capabilities of microfluidic systems by regulating the flow automatically [2]. Beebe *et al*. [2] studied an array of hydrogel-coated posts to control the flow in large channels. The study focused on the response time of the volumetric change of the hydrogel. The prefabricated posts in the channel serve as supports for the hydrogel, thus increasing the stability during swelling and contraction as well as improving the response time due to the short diffusion distance of the hydrogel coat. Eddington and Beebe [16] gave an overview on the advantages of resistance-based valves and hydrogel jacket valves in flow control. Resistance-based valves are made by either patterning an array of hydrogels in a micro channel or patterning two strips of hydrogel along the wall of the micro channels. The pH-sensitive hydrogel has been used as an actuator in micro systems. A hydrogel jacket valve is similar in design to the resistance-based flow control, but it was developed for applications requiring rapid response time.

The hydrogel properties and the underlying mechanisms of the hydrogel’s response to the environment are still not well understood. It is imperative to acquire the mechanical properties and understand the behavior of hydrogels used in micro-fluidic valves. Since hydrogel is a relatively new material, the mechanical performance in responding to stimuli should be well understood before we can make full use of this type of material. Hong *et al*. [10,24] developed inhomogeneous gel deformation theory and showed that the swelling of the network in equilibrium is equivalent to the deformation of a hyperelastic material. Marcombe *et al*. [21] adopted this theory to propose a pH-sensitive hydrogel theory to study the deformation of hydrogel. In their study, the hyperelastic material could be analyzed by adding a material model and implemented it as a user-defined subroutine in the finite element package, ABAQUS. The implementation enables the analyses of diverse and relevant phenomena. 

In order to assimilate the gel material into practical applications, this study conducts the simulation and analysis of the micro-fluidic valve performance for pH-sensitive hydrogel. The inhomogeneous gel deformation theory is adopted to investigate the behavior of the hydrogel used in micro-fluidic valves. The hydrogel sphere has been fabricated inside a steel pipe to act as both a sensing and actuating element. The gel sphere swells or shrinks inside the pipe to regulate the flow through the pipe. 

In this paper, besides covering the modeling and simulation of a pH-sensitive hydrogel with an application as a microfluidic valve, the fluid-structure interaction (FSI) effect on the deformation of the gel valve is also considered to improve the accuracy of the results. With low stiffness, hydrogel will encounter significant deformation while interacting with fluid and this deformation could lead to great changes in pressure distribution on the hydrogel surface. Thus, it is important to investigate the impact of the incoming fluid on the hydrogel sphere. Section 2 discusses the theory of the hydrogel and the implementation for simulation, together with the introduction of the FSI theory. In Section 3, the influence of fluid flow on hydrogel deformation and performance is discussed through simulation. We cover three aspects of results, (i) the gel sphere deformation, (ii) the effect of fluid flow on gel deformation and (iii) the effect of gel sphere deformation on the incoming fluid pressure and velocity. The study on these three aspects demonstrates the effects of fluid flow on the more accurate prediction of gel sphere deformation and in turn on the flow regulations.

## 2. Inhomogeneous Deformation Theory of pH Sensitive Hydrogel

### 2.1. Hydrogel Deformation

**Figure 1:**
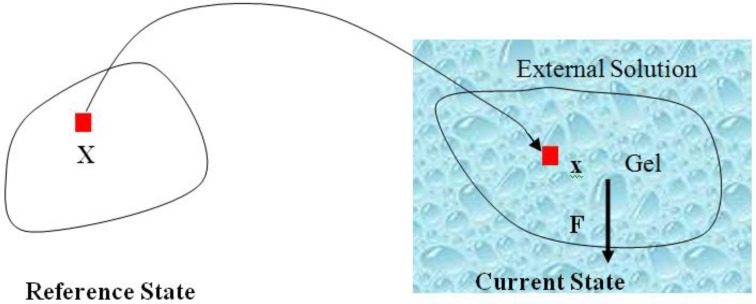
A hydrogel is in contact with an aqueous solution with a fixed chemical potential. A dry network is taken to be the state of reference. For the current state, the network is immersed in an aqueous solution and subjected to a set of mechanical forces (mechanical load and a geometrical constraint).

Consider a network of polymers in contact with a solvent, subject to a mechanical load and geometric constraint, under a constant temperature. The schematic representation is as shown in Figure 1. If we take the stress-free dry network as the reference state, the deformation gradient of the network is defined as:

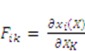
(1)
where, 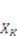 are the network coordinates of a gel system at reference state and 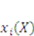 is the network coordinate of a gel system at a current or deformed state [24,25,26].

In the current state, 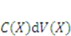 denotes the number of solvent molecules in an elemental volume 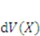 with a solvent concentration in the gel, 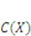. The combination of the two fields 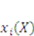 and 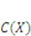 describes the state of the gel system in which the field 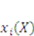 describes the deformation of the network, while 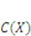 describes the distribution of the solvent molecules in the gel system [24,25]. Let 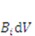 be the external mechanical body force applied on the elemental volume, and 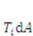 the external mechanical force applied on an elemental area. When the network deforms by a small amount, 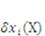, (a small virtual displacement), the field of mechanical load does work 
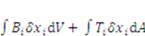
. The integrals extend over the volume and the surface of the network in the reference state. If we assume 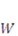 to be the Helmholtz free energy density, the gel free energy in the elemental volume 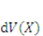 will be 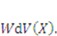 When the field of concentration in the gel changes by 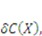 the external solution does work 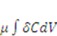, where 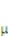 is the chemical potential of the solvent molecules. Thermodynamics dictates that the change in the free energy of the gel should equal the sum of the work done by the external mechanical force and the external solvent:

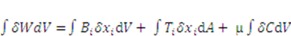
(2)

Thus, the gel is in a state of equilibrium characterized by two fields, 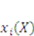 and 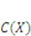. The free energy density of the gel, *W*, is a function of the deformation gradient of the network, **F**, and the concentration of the solvent in the gel, *C*. When the gel equilibrates with the solvent and the mechanical load, the chemical potential, *µ*, of the solvent molecules is homogeneous in the external solvent and in the gel [24]. Hence the chemical potential will be:

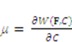
(3)

Introduce a new free-energy function 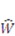 by using a Legendre transformation:

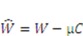
(4)

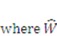
 is a function of the deformation gradient of the network and the chemical potential of the solvent molecules, that is 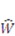 (*F*, *µ*). Combining Equations 2 and 4 gives us:

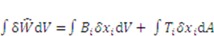
(5)

Equation (5) has the same form as that in solid mechanics. Once the function 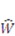 (*µ*) is prescribed, it can be solved via finite element method [24].

The material behavior of hydrogel is analogous to a compressible hyperelastic solid and a material model characterized by a free-energy function developed by Flory and Rehner [27,28], Ricka and Tanaka [29], and Brannon-Peppas and Peppas [30]. In the neutral state with the assumption of incompressibility of individual polymeric and water molecules, the free energy form can be expressed as [24]

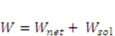
(6)

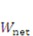
 is due to the network stretch, and 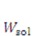 results from the solvent-network blending. The constraint between the deformation field and concentration field can be derived from the assumption of the incompressibility of individual polymeric and water molecules. This constraint is written as 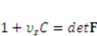, where 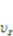 is the volume per solvent molecule [24]. The free energies of stretching the network and mixing the solvent with the network are:

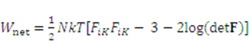
(7)

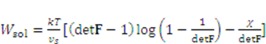
(8)
where 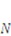 is the number of polymeric chains per reference volumn and 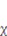 is a dimensionless measure of the enthalpy of mixing. When 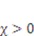, the solvent molecules’ diffusion is less energetically favorable. For pH-sensitive gel, the detailed energy form is derived by Marcombe *et al*. [21]. Following previous work [21,22,23,24], an idealized model is adopted, assuming that the free-energy density of the gel is a sum of several contributions:

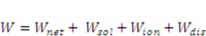
(9)

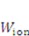
 is the energy due to solvent-ion mixing, and 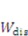 is derive from the acidic group dissociation. 

The state of the inhomogeneously swollen gel is specified by the following independent fields: 
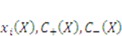
 and 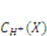, where 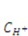 is the nominal concentration of the hydrogen ions, 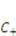 is the nominal concentration of the counter-ion that carries charge of sign opposite to the fixed charge, 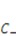 is the nominal concentration of the co-ion that bears a charge of the same sign as the fixed charge. We stipulate that the nominal density of free energy is 
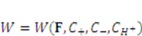
. According to Marcombe *et al*. [21], the concentrations of the mobile ions are taken to be low, so that their contribution to the free energy is due to the entropy of mixing, namely,

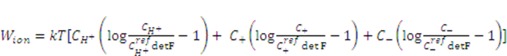
(10)
where 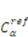 is a reference value of the concentration of 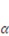 species.

The contribution due to the dissociation of the acidic groups is as follows:

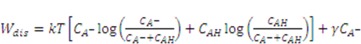
(11)

The expression comprises the entropy and the enthalpy of dissociation, where *γ* is the increase in the enthalpy when an acidic group dissociates. Note that 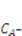 and 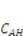 are the nominal concentrations of the fixed charge and of the associated acidic group. They are constrained by 
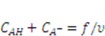
, where 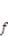 is the ratio between acidic groups on a polymer chain and the total number of monomers on the chain and 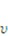 is the volume per monomer. 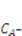 and 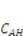 can be expressed using independent fields as 
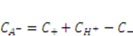
 (electroneutrality) and 
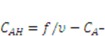
.

The number of particles of species in the gel at the current state divided by the volume of the dry network defines the nominal concentration of the species, *C_a_*. The same number divided by the volume of the gel in the current state defines the true concentration of the species, ***c**_a_*. The two definitions are related, as 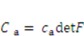. Recall that when the number of particles is counted in units of the Avogadro number, *N_A_* = 6.023 × 10^23^, the molar concentration of the species *α* is designated by [*α*]; for example, 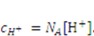


Recall a relation in continuum mechanics connecting the true stress σ_ij_ and the nominal stress 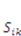: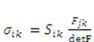, for pH-sensitive hydrogel it can be derived as [21]

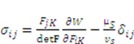
(12)

Using the function 
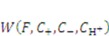
 as specified above, equation (12) becomes

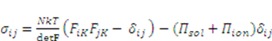
(13)

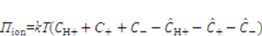
(14)

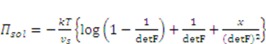
(15)

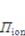
 is the osmotic pressure due to the imbalance of the number of ions in the gel and in the external solution, 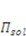 is the osmotic pressure due to the mixing of the network and the solvent. Using the above governing equations, the free-energy function can be coded into subroutine UHYPER for ABAQUS simulation. More complex boundary value problems can also be solved with the same subroutine. 

In numerical calculations, we have assumed that the volume per monomer equals the volume per solvent molecule 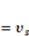, and the following parameters and values are used in the simulation. The number *f* is the number of acidic groups on a polymer chain divided by the total number of monomers on the chain, *f* = 0.05; 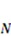 is the number of polymer chains per unit volume of the dry network, *v* is the volume per monomer, 1/*Nv* is the number of monomers per polymer chain, and *Nv* = 0.001; *χ* is a dimensionless measure of the enthalpy of mixing, an indicator of the ease of polymer-solvent interaction, *χ* = 0.1; dissociation constant p*K_a_* = −log_10_*K_a_* = 4.3, initial stretch 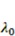 = 3.39; 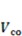 is the concentration in external solution, 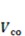 = 6.02 × 10^−5^. Besides these basic parameters, the following parameters are involved: *kT* is the temperature in the unit of energy, *kT* = 4 × 10^−21^ J and *kT*/*v* = 4 × 10^7^ Pa at room temperature. A representative value of the volume per molecule is *v* = 10^−28^ m^3^. The elastic modulus of the dry network is *NkT*. For *Nv* = 10^−3^, the elastic modulus is *NkT* = 4 × 10^4^ Pa. These values are adopted to study the behavior of hydrogel as a microfluidic valve elaborated in section 3.

### 2.2. Fluid-Structure Interaction Models of Hydrogel and Fluid

Fluid-structure interactions (FSI), the interactions of deformable structures with a surrounding fluid flow, have significant effects on modeling and computational issues. It is also a challenging multi-physics problem. In earlier micro-fluidics study, the gel material was regarded as a solid structure for micro-fluidic valves. Since gel material is very soft, it can easily deform under pressure, and FSI should be considered for the design of gel valve. In the present simulation, the evaluation of fluid flow coupled with the function of the gel valve structure when it swells are addressed. In the coupling system, the nonlinear gel deformation has been carried out by using a finite elements method through commercial software ABAQUS and gel subroutine. The governing equations for incompressible viscous fluid flow under an Arbitrary Lagrangian-Eulerian (ALE) coordinate system can be formulated as follows:

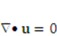
(16)

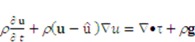
(17)

In the above-mentioned continuity and momentum equations, 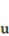, 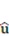 and 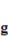 denote the fluid velocity, grid velocity and gravitational acceleration respectively. The stress tensor 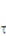 is defined as 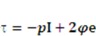, and **e** denotes the velocity strain tensor, which is in the form of 
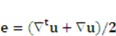
. In these formulae, *p* and 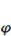 denote pressure and dynamic viscosity respectively.

The kinematic and dynamic conditions applied to the fluid-structure interface are as follows:

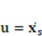
(18)

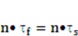
(19)
where 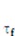 denotes the fluid stresses, and 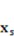 and 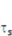 denotes the solid gel displacement and stress respectively at the FSI interface. 

It should be noted that the moving boundary conditions are updated with the latest structural displacements at the interface. The movement of boundary nodes may reduce the mesh quality, resulting in lower computational efficiency and accuracy. Thus, it becomes necessary to adjust the interior nodes to keep good mesh quality. To achieve this, we solve the following Laplace Equation:

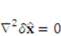
(20)
where 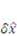 denotes the increment of the displacement. The latest displacement is then updated by adding the incremental solutions. 

A finite element method has been employed to solve the Laplace equation, based on either the latest nodal positions or the initial nodal positions. In the simulation, either two-way coupling method or a one-way coupling method can be used to solve FSI problem. To simplify the process, we use a one-way FSI coupling approach in this study. FLUENT and ABAQUS are used to solve gel FSI problems. At each stage, the deformed configuration of the gel valve under a particular pH value is used to initiate the configuration for computational fluid dynamic (CFD) simulation to obtain the corresponding flow pattern inside the pipe, together with the pressure distribution on the gel valve. In this study, FLUENT was used to perform CFD simulation. The pressure field obtained from CFD is then applied as boundary conditions on to the hydrogel in ABAQUS to calculate the deformation of the gel valve. The deformed geometry will be used as input for CFD simulation to calculate the pressure on the deformed gel valve, which will be used in the mechanical simulation to get further deformation of the gel. We iterate between the fluid flow (CFD) and gel deformation simulations until differences at each step are negligible. 

## 3. Results and Discussion

### 3.1. Free Swelling of pH-sensitive Hydrogel

**Figure 2:**
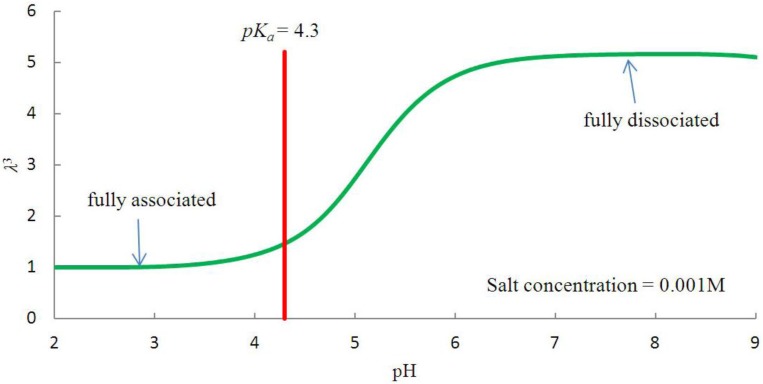
The variation of swelling ratio as a function of pH values for a fixed salt concentration.

To investigate the swelling behavior of pH-sensitive hydrogel as a micro-fluidic valve, the free swelling ratio as a function of pH at a fixed salt concentration is studied via inhomogeneous gel theory [21]. Figure 2 shows the free swelling ratio of gel volume as a function of pH at a fixed salt concentration. The hydrogel remains steady at low pH values and swells at pH values higher than the p*K_a_* value. This pH-induced swelling behavior can be attributed to the presence of acidic groups bounded to the polymer network, and two limits in Figure 2 should be emphasized: (i) fully associated limit and (ii) fully dissociated limit with respect to the p*K_a_* value.

The swelling of the hydrogel is marginal as pH value varies below p*K_a_.* However, there is a slight increase as the pH value approaches p*K_a_*. The hydrogel expands at a much greater rate when the pH value is greater than the p*K_a_* value, as the osmotic force induces the hydrogel to swell for pH ranging between 4 and 7. When the ionization process reaches its saturation point, a further increase in pH value does not affect the swelling behavior of the hydrogel. This can be observed in Figure 2 where the hydrogel ceases to swell when pH > 7.

### 3.2. Inhomogeneous Swelling of Hydrogel as Microfluidic Valve

**Figure 3:**
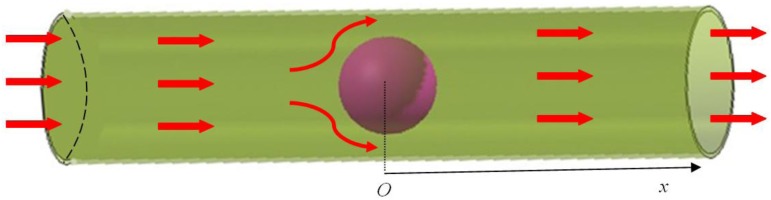
A spherical hydrogel as a micro-fluidic valve controlling the fluid flow in the pipe.

To study the mechanical behavior of hydrogel used as a microfluidic valve, the valve structure is constructed as shown in Figure 3. In this assembly, a spherical hydrogel inside the cylindrical pipe is considered. The hydrogel is coated on a solid particle of radius 0.25 mm. The latter is used to hold the hydrogel in place, creating the microfluidic valve from pH-sensitive hydrogel to regulate the flow of an aqueous solution in the pipe. The spherical hydrogel (excluding solid core) has an initial thickness of 0.75 mm coated on the core. The pipe is made of hard material such as steel with an inner radius of 1.7 mm. The fluid flows through the pipe that can also be used to constrain the swelling of the hydrogel. The thickness of the steel pipe is less relevant as we can regard the steel pipe as a rigid body when compared with the soft hydrogel sphere. According to free swelling properties of pH-sensitive hydrogel, the hydrogel sphere will expand at high pH value of the external solution to block the flow and shrink at low pH value to ease the flow. The hydrogel sphere will swell until it touches the inner surface of the pipe to block the flow of fluid. The schematic representation of a hydrogel valve assembly is shown in Figure 3. 

Marcombe *et al.* [21] simulated a similar case of the hydrogel coated on a rigid pillar in a microfluidic channel. They observed the same swelling behavior to regulate the flow but their study did not consider the flow influence on the soft hydrogel deformation (FSI effects). There is an array of the same type of hydrogel posts in a channel; the hydrogel coated on the perimeter will expand at high pH to block the channel. At a lower pH value, the hydrogel shrinks to allow the fluid to pass the microfluidic valve [2]. So far, several studies on hydrogel microfluidic valves have been carried out, but the studies did not consider the FSI effects on hydrogel deformation. Under the FSI condition, the spherical hydrogel will not deform homogeneously upon exposure to a pH value in the environment. FSI occurs when a fluid interacts with the soft hydrogel and exerts pressure on it. This normally causes further deformation in the hydrogel valve altering the fluid flow. As the hydrogel is very soft, it is crucial to consider FSI effects in the design of the hydrogel microfluidic valve, and hence we have performed the study of swelling behavior of the hydrogel with respect to pH values and FSI effects. The solid gel deformation, fluid pressure distribution and fluid velocity distribution are investigated for microfluidic valve with and without FSI effects for comparison. We assume the inlet fluid pressure to be 2000 Pa.

The deformed radii of the spherical gel considered the increase in pH value without FSI effects at various pH values are tabulated in Table 1. We select six particular outer radii of the gel, based on its original outer radius of 1mm and the constraint pipe with a radius of 1.7 mm. The imposed inlet pressure induces pressure on the hydrogel sphere surface which alters further the free swelling shape of the spherical gel. To study the effects of FSI on the gel valve, the deformed configurations of the gel at the above six representative stages are simulated. 

**Table 1:** Data collection for hydrogel valve with and without FSI effects at six stages

Stages	Six stages	Effective radius(mm)		Velocity		FAR
		Without FSI	With FSI	Without FSI	With FSI	
1	pH = 4.15	1.100	1.125	1.30	1.15	3.3%
2	pH = 4.55	1.200	1.235	1.25	1.05	5.9%
3	pH = 4.80	1.300	1.335	1.05	0.80	7.7%
4	pH = 5.05	1.400	1.450	0.80	0.55	15.3%
5	pH = 5.30	1.500	1.554	0.50	0.25	25.8%
6	pH = 5.60	1.600	1.647	0.20	0	46.2%

**Figure 4:**
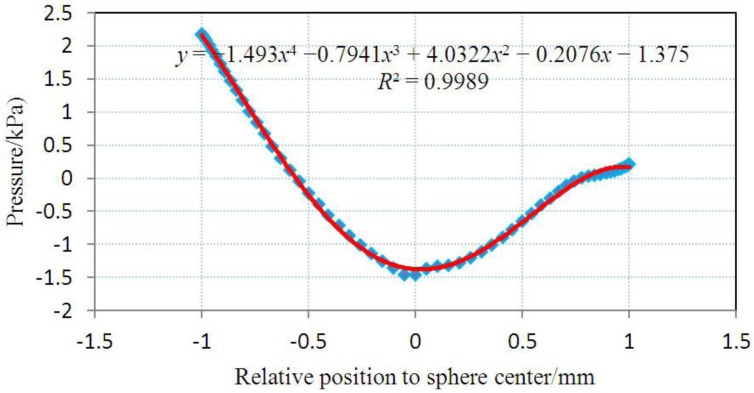
The distribution of surface pressure with an inlet pressure of 2000 Pa.

**Figure 5:**
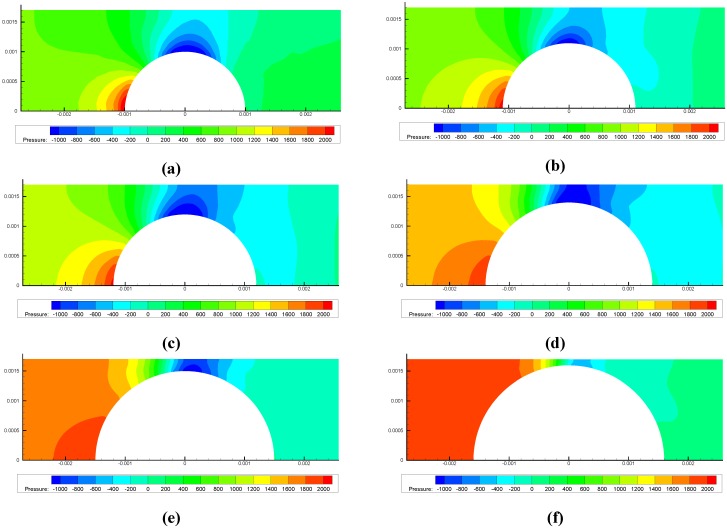
Pressure distribution patterns at various stages with an inlet pressure of 2,000 Pa. (**a**) for stage 1; (**b**) for stage 2; (**c**) for stage 3; (**d**) for stage 4; (**e**) for stage 5 and (**f**) for stage 6.

At each stage, we provide the pH value with the corresponding deformed configuration of the gel valve, the pressure and the velocity caused by the FSI. Owing to the cylindrical symmetry, the 3-D model can be simplified to a 2-D axis symmetrical model, and we can obtain the pressure on the surface of the hydrogel by simulating fluid flow in the pipe using FLUENT. Figure 4 presents the pressure on the surface of the gel as a function of *x* at the original state with an inlet pressure of 2,000 Pa. The pressure values obtained on the gel surface are imposed as the boundary conditions to the hydrogel in ABAQUS causing additional gel deformation. We iterate between the fluid flow and gel deformation simulations until differences at each step are negligible. The pressure patterns at various stages are illustrated in Figure 5.

**Figure 6:**
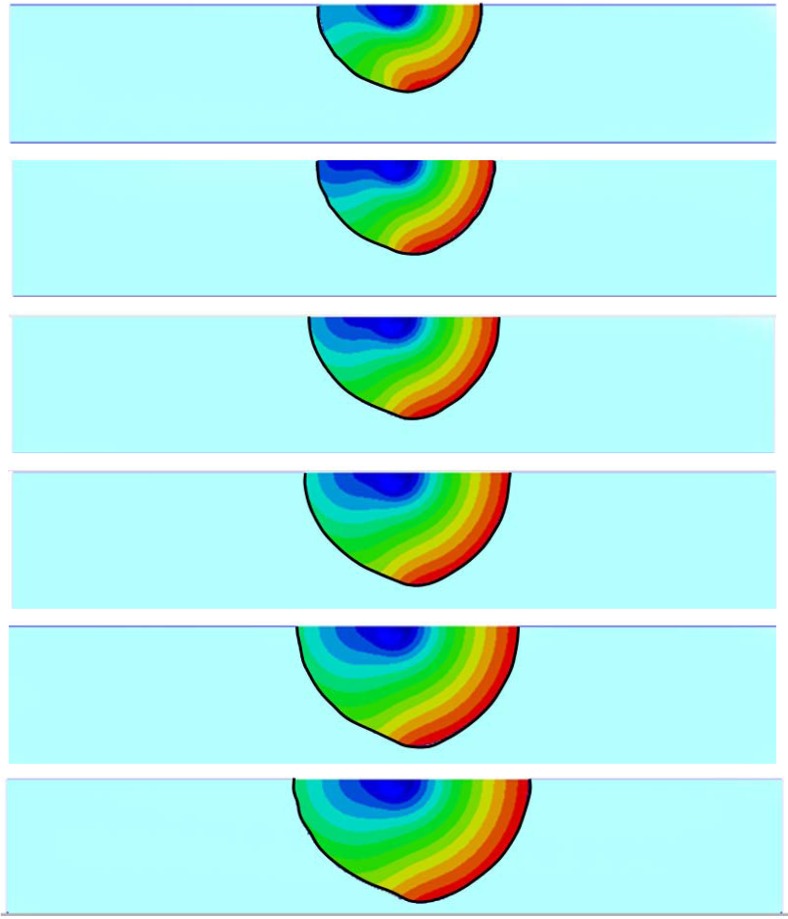
The deformation contour plots of hydrogel under the inlet pressure of 2,000 Pa considering FSI for six different spherical gel initial radii. (Half spherical section view).

Figure 6 illustrates the deformation shapes and contours of the hydrogel at various stages with the 2,000 Pa inlet pressure. 

**Figure 7:**
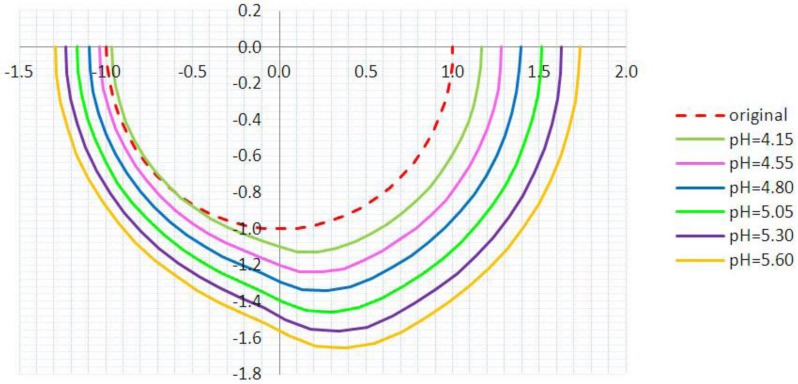
The shapes of the hydrogel at various stages with an inlet pressure of 2000 Pa. (Half spherical section view).

The deformed configurations of the hydrogel at six selected stages are depicted in Figure 7. The original hydrogel sphere expanded homogeneously along the radius direction without FSI, whereas the shape of the hydrogel sphere is distorted once FSI is considered in the analysis. The shape variations at various pH values can be observed in Figure 7, which compares the deformed configurations of the hydrogel at these six stages.

**Figure 8:**
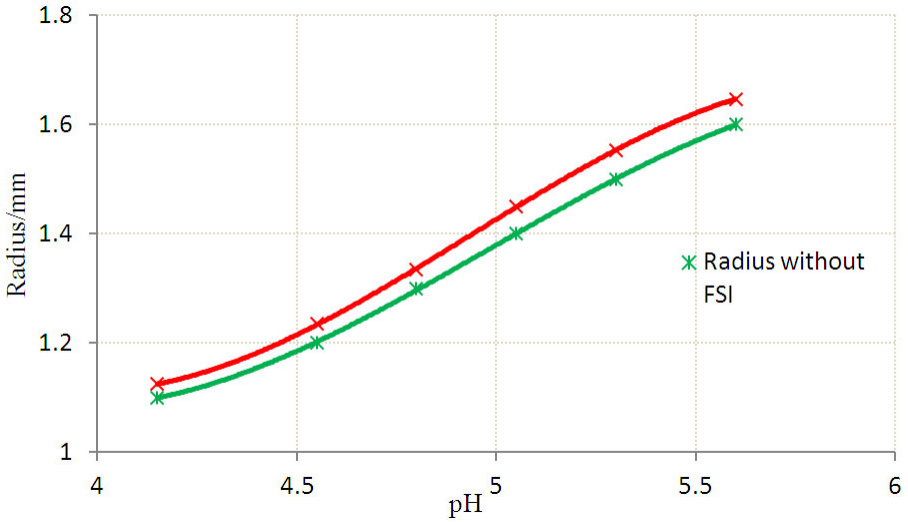
The equivalent radius with FSI or without FSI at various stages.

**Figure 9:**
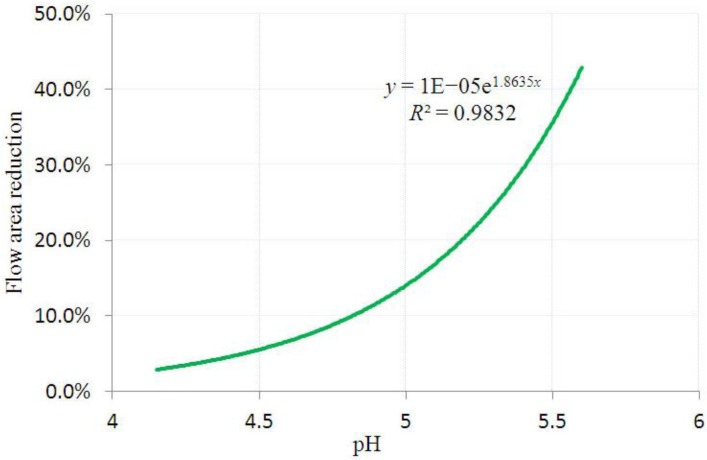
The flow area reduction due to the fluid pressure at various pH values.

The deformation of the hydrogel due to FSI increases the effective blocking area. The effective radii of the blocking area corresponds to the maximum vertical distance from the gel centroid, as shown in Figure 7. The effective radii obtained from the analyses with and without FSI are compared in Figure 8. The flow area reduction (FAR) is defined as the ratio between the reduced cross-sectional area due to FSI and the non-pressurized flow area. The variation of FAR at various pH values is presented in Figure 9.

As shown in Figure 9, the flow area reduction follows an exponential relationship with the solvent pH value, which implies that FSI effects on FAR increase more rapidly as the pH value approaches the fully dissociated value. If we let the flow area reduction be 1, the pH value is approximately 6.18. At this particular pH value, solvent can still flow through the pipe, without considering FSI effects. However, this is not actually possible when we predict the deformed configurations of the hydrogel taking FSI into consideration. 

**Figure 10:**
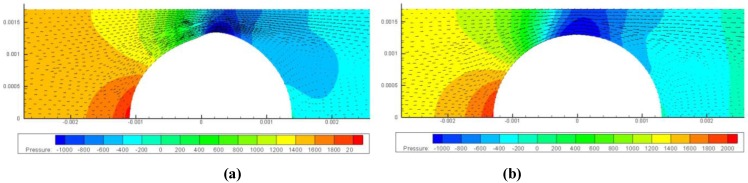
The pressure contour and velocity plot under pressure of 2,000 Pa at 1.3 mm. (**a**) Gel radius = 1.3 mm velocity contour without FSI; (**b**) Gel radius = 1.3 mm velocity contour considering FSI.

**Figure 11:**
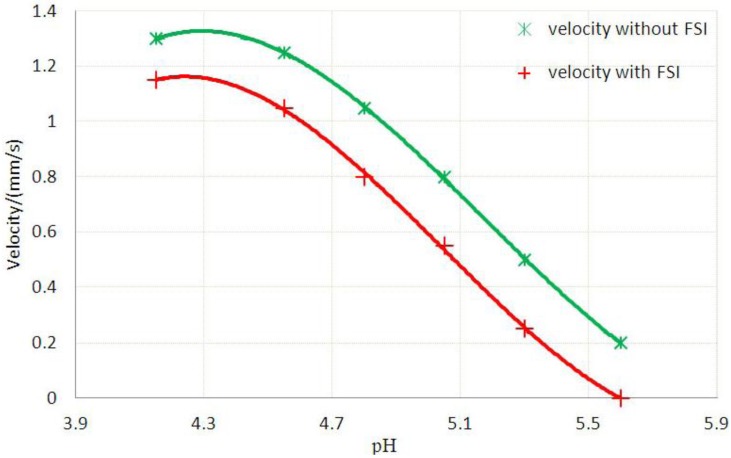
Maximum velocities of flow at various stages with or without FSI.

The velocity distribution is also affected by FSI and consequently the flow rate. We can simulate the velocity distribution at various stages and calculate the overall flow rate via a surface integration over the cross-sectional area. The velocity distribution at pH = 4.8 is shown in Figure 10 and the maximum velocities of flow at various stages with and without FSI are shown in Figure 11.

Table 1 also indicates detailed results collected at various pH values with and without FSI effects. Significant increases in FAR values are observed at higher pH values, which imply more significant effects of FSI at higher pH values. The valve blocks the flow nearly completely at pH value of 5.60. The findings demonstrate that FSI effects have to be considered in the design of hydrogel for microfluidic valves.

## 4. Concluding Remarks

In our study, a numerical simulation on pH-sensitive hydrogel embedded in a long micro pipe which takes fluid structure interaction (FSI) into account has been carried out. The investigation covers the deformation configurations of the hydrogel and velocity distribution patterns at various pH values that influence significantly the swelling ratios of the gel in the range 4.15 to 5.60. The inspiring results provide an insight into the structural response of the hydrogel which acts as a microfluidic valve to control fluid flow in a micro pipe. With reference to the variation of the values of flow area reduction (FAR), we observe a significant reduction in flow area when FSI is incorporated in the calculation. Therefore, it is essential to include FSI in the analyses for more accurate design and fabrication of the hydrogel micro-fluidic flow control apparatus. The results also demonstrate that the inhomogeneous gel theory with FSI can be used to study pH-sensitive gel valve swelling deformation with higher accuracy. However, in the present study, we eliminate the diffusion process and assume a homogeneous concentration distribution at the selected stages. The response time constant will be taken into account in further study to improve the applicability of our model.

## References

[1] Beebe D.J., Mensing G.A., Walker G.M. (2002). Physics and applications of microfluidics in biology. Annu. Rev. Biomed. Eng..

[2] Beebe D.J., Moore J.J.S., Bauer J.M., Yu Q., Liu R.H., Devadoss C., Jo R.-H. (2000). Functional hydrogel structures for autonomous flow control inside microfluidic channels. Nature.

[3] Hoffmann J., Plötner M., Kuckling D., Fischer W.J. (1999). Photopatterning of thermally sensitive hydrogels useful for microactuators. Sens. Actuat. A.

[4] Wang J., Chen Z., Mauk M., Mauk M., Hong K.S., Li M., Yang S., Bau H.H. (2005). Self-actuated, thermo-responsive hydrogel valves for lab on a chip. Biomed. Microdevices.

[5] Osada Y., Rossmurphy S.B. (1993). Intelligent gels. Sci. Amer..

[6] Baldi A., Gu Y., Loftness P.E., Siegel R.A., Ziaie B. (2003). A hydrogel-actuated environmentally sensitive microvalve for active flow control. J. Microelectromech. Syst..

[7] Richter A., Paschew G., Klatt S., Lienig J., Arndt K.-F., Adler H.-J.P. (2008). Review on hydrogel-based pH sensors and microsensors. Sensors.

[8] Seitz W.R., Rooney M.T.V., Miele E.W., Wang H., Kaval N., Zhang L., Doherty S., Milde S., Lenda J. (1999). Derivatized, swellable polymer microspheres for chemical transduction. Anal. Chim. Acta..

[9] De S.K., Aluru N.R. (2004). A chemo-electro-mechanical mathematical model for simulation of pH sensitive hydrogels. Mech. Mater..

[10] Hong W., Zhao X.H., Zhou J.X., Suo Z.G. (2008). A theory of coupled diffusion and large deformation in polymeric gels. J. Mech. Phys. Solids.

[11] Seigel R.A. (1991). Implantable, Self-Regulating Mechanochemical Insulin Pump.

[12] Shahinpoor M. (1995). Microelectromechanics of ionic polymeric gels as electrically controllable artificial muscles. J. Intell. Mater. Syst. Struct..

[13] Chen G.P., Takashi U., Tetsuya T. (2002). Scaffold design for tissue engineering. Macromol. Biosci..

[14] Chan A.W., Neufeld R.J. (2009). Modeling the controllable ph-responsive swelling and pore size of networked alginate based biomaterials. Biomaterials.

[15] Dong L., Jiang H.R. (2007). Autonomous microfluidics with stimuli-responsive hydrogels. Soft matter..

[16] Eddington D.T., Beebe D.J. (2004). Flow control with hydrogels. Adv. Drug Rev..

[17] Yi C.Q., Li C.W., Ji S.L., Yang M.S. (2006). Microfluidics technology for manipulation and analysis of biological cells. Anal. Chim. Acta..

[18] Johnson B., Niedermaier D.J., Crone W.C., Moorthy J., Beebe D.J. Mechanical properties of a pH sensitive hydrogel. Proceedings of the 2002 Society for Experimental Mechanics (SEM) Annual Conference.

[19] Zhao B., Moore J.S. (2001). Fast pH and ionic strength-responsive hydrogels in microchannels. Langmuir.

[20] Kurnia J.C., Erik-Birgersson E, Mujumdar A.S. (2011). Computational study of pH-sensitive hydrogel-based microfluidic flow controllers. J. Funct. Biomater..

[21] Marcombe R., Cai S., Hong W., Zhao X., Lapusta Y., Suo Z. (2010). A theory of constrained swelling of a pH-sensitive hydrogel. Soft Matter..

[22] De S.K., Aluru N.R., Jhonson B, Crone W.C., Beebe D.J., Moore J. (2002). Equilibrium swelling and kinetics of pH-responsive hydrogels: Models, experiments and simulations. J. Microelectromech. Syst..

[23] Hu Y.H., You J.O., Auguste D.T., Suo Z., Vlassak J.J. (2012). Indentation: A simple, nondestructive method for characterizing the mechanical and transport propertie of pH-sensitive hydrogels. J. Mater. Res..

[24] Hong W., Liu Z.S., Suo Z.G. (2009). Inhomogeneous swelling of a gel in equilibrium with a solvent mechanical load. Int. J. Solids Struct..

[25] Liu Z.S., Hong W., Suo Z.G., Swaddiwudhipong S., Zhang Y.W. (2010). Modeling and simulation of buckling of polymeric membrane thin film gel. Comput. Mater. Sci..

[26] Liu Z.S., Swaddiwudhipong S., Cui F.S., Hong W., Zhang Y.W. (2011). Analytical solutions of polymer gel structures under buckling and wrinkle. Int. J. Appl. Mech..

[27] Flory P.J., Rehner J. (1943). Statistical mechanics of cross-linked polymer networks I. Rubber elasticity. J. Chem. Phys..

[28] Flory P.J., Rehner J. (1943). Statistical mechanics of cross-linked polymer networks II. Swelling. J. Chem. Phys..

[29] Ricka J., Tanaka T. (1984). Swelling of ionic gels: Quantitative performance of the Donnan Theory. Macromolecules.

[30] Brannon-Peppas L., Peppas N.A. (1991). Equilibrium swelling behavior of dilute ionic hydrogels in electrolytic solutions. Chem. Eng. Sci..

